# Application of immune enhanced organoids in modeling personalized Merkel cell carcinoma research

**DOI:** 10.1038/s41598-022-17921-6

**Published:** 2022-08-16

**Authors:** Steven D. Forsythe, Richard A. Erali, Preston Laney, Hemamylammal Sivakumar, Wencheng Li, Aleksander Skardal, Shay Soker, Konstantinos I. Votanopoulos

**Affiliations:** 1 Wake Forest Institute for Regenerative Medicine, Wake Forest School of Medicine, Winston-Salem, NC USA; 2 Department of Cancer Biology, Wake Forest School of Medicine, Winston-Salem, NC USA; 3 Wake Forest Organoid Research Center (WFORCE), Winston Salem, USA; 4 Department of Surgery, Division of Surgical Oncology, Wake Forest Baptist Health, Wake Forest University, Medical Center Boulevard, Winston Salem, NC 27157 USA; 5 Wake Forest Comprehensive Cancer Center, Wake Forest School of Medicine, Winston Salem, USA; 6 Department of Biomedical Engineering, The Ohio State University, Columbus, OH USA; 7 Department of Pathology, Wake Forest School of Medicine, Winston-Salem, NC USA; 8 The Ohio State University and Arthur G. James Comprehensive Cancer Center, Columbus, OH USA

**Keywords:** Cancer models, High-throughput screening

## Abstract

Merkel cell carcinoma (MCC) is a rare neuroendocrine cutaneous cancer, with incidence of less than 1/100,000, low survival rates and variable response to chemotherapy or immunotherapy. Herein we explore the application of patient tumor organoids (PTOs) in modeling personalized research in this rare malignancy. Unsorted and non-expanded MCC tumor cells were isolated from surgical specimens and suspended in an ECM based hydrogel, along with patient matched blood and lymph node tissue to generate immune enhanced organoids (iPTOs). Organoids were treated with chemotherapy or immunotherapy agents and efficacy was determined by post-treatment viability. Nine specimens from seven patients were recruited from December 2018-January 2022. Establishment rate was 88.8% (8/9) for PTOs and 77.8% (7/9) for iPTOs. Histology on matched patient tissues and PTOs demonstrated expression of MCC markers. Chemotherapy response was exhibited in 4/6 (66.6%) specimens with cisplatin and doxorubicin as the most effective agents (4/6 PTO sets) while immunotherapy was not effective in tested iPTO sets. Four specimens from two patients demonstrated resistance to pembrolizumab, correlating with the corresponding patient’s treatment response. Routine establishment and immune enhancement of MCC PTOs is feasible directly from resected surgical specimens allowing for personalized research and exploration of treatment regimens in the preclinical setting.

## Introduction

Merkel cell carcinoma (MCC) is a rare and aggressive neuroendocrine cutaneous cancer with an estimated annual incidence of 0.7 cases per 1,000,000^1^ and worse survival outcomes than melanoma. While early research suggested MCC arose from neural crest origin, more recent evidence points to epidermal progenitors^2,3^. Malignant transformation results as a sequela of infection with the Merkel cell polyomavirus (MCPyV) in approximately 60-80% of MCC cancers, while in a smaller subset of patients is linked to UV exposure^4^, with the two events synergistically increasing the mutational load and neoantigenicity of MCC tumors.

Most patients with MCC present with loco-regional disease which requires surgical resection followed by adjuvant radiation in selected patients^4^. In patients with metastatic disease, there are limited options while the rarity of MCC makes it difficult to accrue patients for comparative studies with new agents^5,6^. There is a rapidly growing interest in check point inhibitors as well as in the development of a MCPyV vaccine^7–9^. However, despite promising results with immunotherapy that resulted in FDA approval in 2017, as with many other cancers, acquired tumor resistance remains a serious problem along with immunotherapy related toxicity^10^. Thus, predicting potential treatment failure is important in avoiding treatment related adverse effects in cases where a survival benefit is not expected.

Due to the low incidence for MCC, alternative options to clinical trials are required to determine preclinical treatment efficacy. We have previously reported successful organoid modeling of several tumor types, including mesothelioma, lung, appendiceal, melanoma, sarcoma, and colorectal primaries^11–16^. Importantly, some of these studies analyzed rare tumor subtypes which have seen little advancement in clinical management over the past few decades. These organoids were successfully created in a highly controlled hydrogel system comprised of collagen and hyaluronic acid, eliminating the necessity for xenogeneic basement membrane extract for culture. The PTOs were ready for preclinical testing within 1 week of fabrication, providing treatment data within a timeframe relevant to potentially support and inform clinical decisions. These studies were able to demonstrate utility potential in precision medicine applications for both approved and investigational therapies^12,15,17,18^.

Herein we display the feasibility of these approaches in developing PTOs to create a personalized clinical research platform for MCC at the level of the individual patient. We demonstrate the biofabrication of MCC PTOs from resected patient tissues and show similarity in tissue markers for the tumors. We analyzed the use of both investigational chemotherapy regimens and approved immunotherapy treatments for comparative efficacy. Finally, we were able to compare the results of temporally distinct resections from the same patient with their clinical outcome. We believe the data presented showcases the utility of PTOs in MCC research, and in the future, potentially as a preclinical companion tool in rare malignancies.

## Results

### Patient accrual and workflow summary

**Figure 1:**
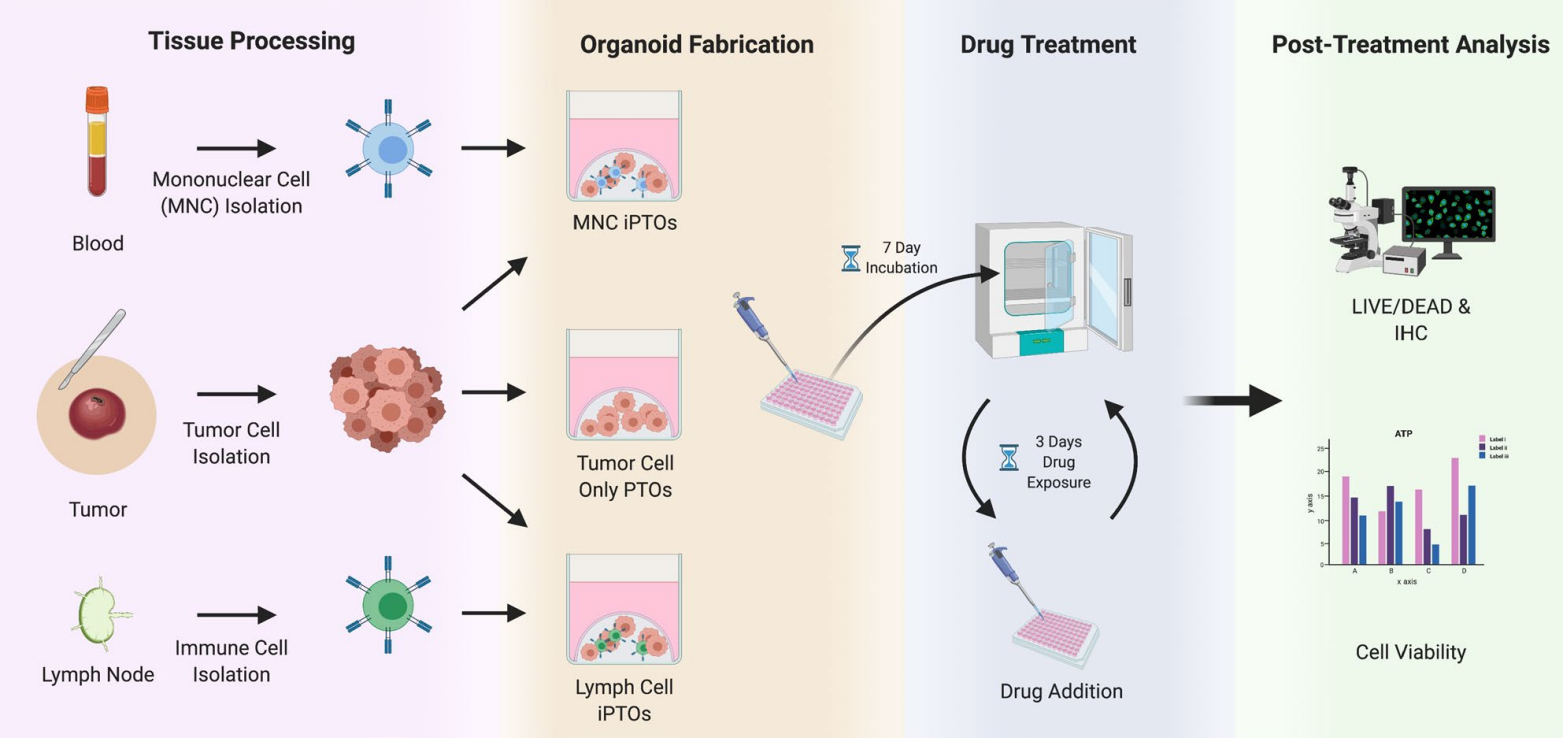
Flow chart of Merkel cell carcinoma organoid fabrication. Tumor cells were isolated from digested tumor tissue and fabricated into PTOs, while immune cells were isolated from patient blood or from lymph tissue and combined with tumor cells to create iPTOs. These organoids were cultured for 1 week, after which they were characterized and screened for therapeutic efficacy. Created with BioRender.com.

Nine tumor specimens from seven patients were acquired during the study period from December 2018–January 2022 (Table 1). Eight of the tumors provided enough cells to produce organoids for therapy screening. One patient underwent a second and third operation for recurrent disease while on immunotherapy and both additional specimens were processed into organoids. Additionally, seven tissues provided sufficient tumor cells with separate specimens for lymphocytes for parallel immunotherapy testing, with six sets using patient blood and one set using a resected lymph node (Fig. 1). After biofabrication, PTOs were cultured for 7 days and underwent 3 days of drug testing. Overall, drug screen results for all specimens were available within 10 days eliminating the need for cell expansion and generating patient-specific data within a clinically relevant timeline.

**Table 1:** Patient demographics, prior treatments, tumor characteristics, and PTO correlation.

Tumor ID	Resected tumor location	Prior therapy	Adjuvant treatment(s)	iPTO component	Immunotherapy?	Current clinical status	PTO correlation with clinical response
MCC1	Neck	None	Radiation	LN	No	Deceased	N/A
MCC2, 7, 9	Face	Surgery	Radiation, immunotherapy	PBMCs^b^	Pembrolizumab	Alive	Yes, lack of responses
MCC3	Face	None	Immunotherapy	PBMCs	Pembrolizumab	Deceased	Yes, lack of responses
MCC4^a^	Arm	None	None	PBMCs	No	Deceased	N/A
MCC5	Face	Surgery	None	None	No	Alive	N/A
MCC6	Ear Canal	None	None	PBMCs	No	AWD	N/A
MCC8	Scalp	None	None	PBMCs	No	NED	N/A

MCC2, 7 and 9 were from the same patient.

*AWD* alive with disease, *LN* lymph node, *PBMCs* peripheral blood mononuclear cells, *NED* no evidence of disease, *N/A* not applicable.

^a^Only tumor where PTOs were not successfully fabricated.

^b^All 3 tumors iPTO immune components were from blood.

### PTOs histologically resemble resected tissue

**Figure 2:**
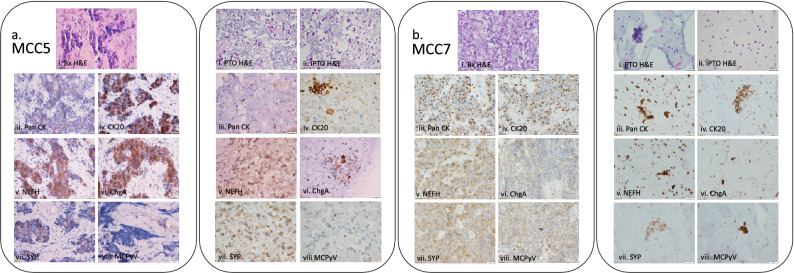
Immunohistochemistry of matched patient tissue (Bx) and Merkel cell carcinoma PTO set (**a**) MCC5 and (**b**) MCC7. Comparison of tissue and PTOs shows high concordance of identification markers including (i) cellular morphology (H&E), (ii) iPTO cellular morphology (H&E), (iii) pan cytokeratin (Pan CK), (iv) cytokeratin 20 (CK20), (v) neurofilament (NFH), (vi) chromogranin A (ChgA), (vii) synaptophysin (SYP and (viii) Merkel cell polyomavirus (MCPyV). Additionally, sets MCC5 (**a**) and MCC7 (**b**) demonstrated variable expression of markers related to disease identification and potential outcomes, including MCPyV. All images taken at 40 × magnification with scale bar 20 μM.

Maintenance of primary tumor cells in organoid culture is essential for accurate tissue representation and proper response to therapeutic intervention. Comparative staining of resected tissue and PTOs demonstrated a high level of correspondence for identifying stains (Fig. 2 and Supplemental Fig. S1). Similar cellular morphology is exhibited in matched patient tissue and PTOs, while iPTOs demonstrated immune cell populations (Fig. 2a,b, i and ii). Positive expressions of Pan-Cytokeratin and CK20 are commonly used to differentiate between MCC from other tumor origins, including melanoma and other metastatic neuroendocrine tumors (Fig. 2a,b, iii and iv)^19^. Gaining its name from the Merkel cell sensory neuron lineage from which it may be derived, MCC also demonstrates positive expression of neural markers including synaptophysin, neurofilament and chromogranin^20^. These markers are also shown to have high correlation between matched PTO and tissue (Fig. 2a, b, v–vii). Additionally, Merkel cell polyomavirus (MCPyV) is hypothesized to play a significant role in development of the disease, as many as 80% of patient cases have virus present. We observed positive expression in 5 of 7 (71%) patient tumors (Fig. 2a,b, viii). IHC staining of PTOs and tissue demonstrated lack of expression for CD45 and S100, eliminating alternative diagnoses that morphologically simulate MCC including lymphoma and melanoma, respectively (Supplemental Fig. S2).

### PTO drug screens allow for regimen optimization

**Figure 3:**
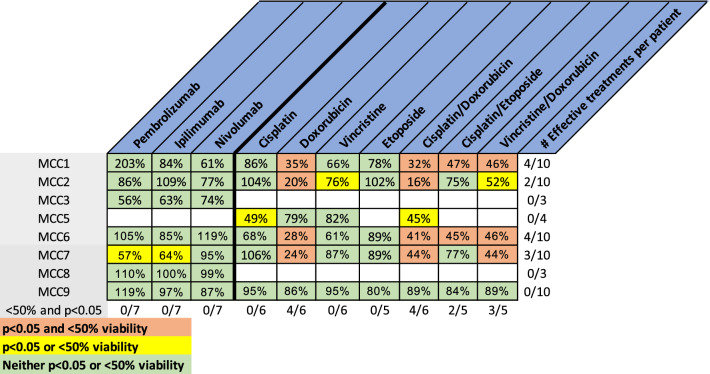
Chemotherapy screening of PTO demonstrates intertumor heterogeneity in tumor treatment response. Percentages are viability compared to patient matched control organoids. Doses for treatments in table are as follows: cisplatin (10 uM), doxorubicin (1 μM), vincristine (1 μM), etoposide (1 μM), cisplatin/doxorubicin (10 μM/1 μM), cisplatin/etoposide (10 μM/1 μM), and vincristine/doxorubicin (1 μM/1 μM). Green boxes represent treatments resulting in < 50% viability decrease and p > 0.05, yellow boxes > 50% or p < 0.05, red boxes represent treatments with > 50% viability decrease and p < 0.05. The bottom line is number of PTO sets in which the treatment is effective (< 50% viable and p < 0.05). The column on the far right represents the number of effective treatments in the respective patient organoid set.

**Figure 4:**
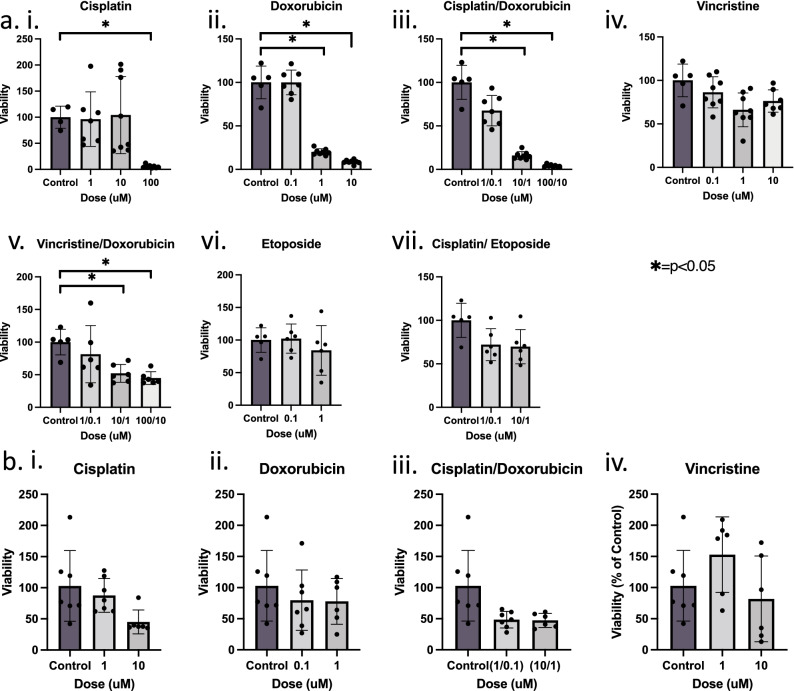
Application of personalized chemotherapy treatment platform for (**a**) MCC2 and (**b**) MCC5 with (i) Cisplatin. (ii) Doxorubicin. (iii) Vincristine. (iv) Etoposide. (v) Cisplatin/Doxorubicin. (vi) Cisplatin/Etoposide and (vii) Vincristine/Doxorubicin. Summary of CellTiter-Glo 3D assay results for (**a**) MCC2 demonstrate dose-dependent treatment responses for (i) Cisplatin (ii) Doxorubicin (iii) Cisplatin/Doxorubicin and (vii) Vincristine/Doxorubicin while (**b**) MCC5 illustrated these results for (i) cisplatin and (iii) cisplatin/doxorubicin. Under each dose, replicates listed as data points. *p < 0.05.

To confirm the feasibility of PTOs in therapeutic screening, PTOs were treated with a series of chemotherapies currently used in the clinical setting (Figs. 3, 4). Overall, therapy response varied between PTOs and treatments, with the most effective regimen being a combination of cisplatin and doxorubicin (4/6, 66%). Analysis of combinational regimens can possibly help to determine the effective components in each regimen on a patient per patient basis or design new therapeutic protocols. For example, PTO set MCC2 displayed significant response towards the combination of cisplatin with doxorubicin, but further analysis of individual response shows the response is not synergistic but mostly due to doxorubicin cytotoxicity (Figs. 3, 4ai–iii). In contrast, PTO set MCC5 used the same combination, but individual response suggests cisplatin is responsible for the regimen’s efficacy (Figs. 3, 4i–iii).

### iPTO platform allows for comparative testing of immunotherapy

**Figure 5:**
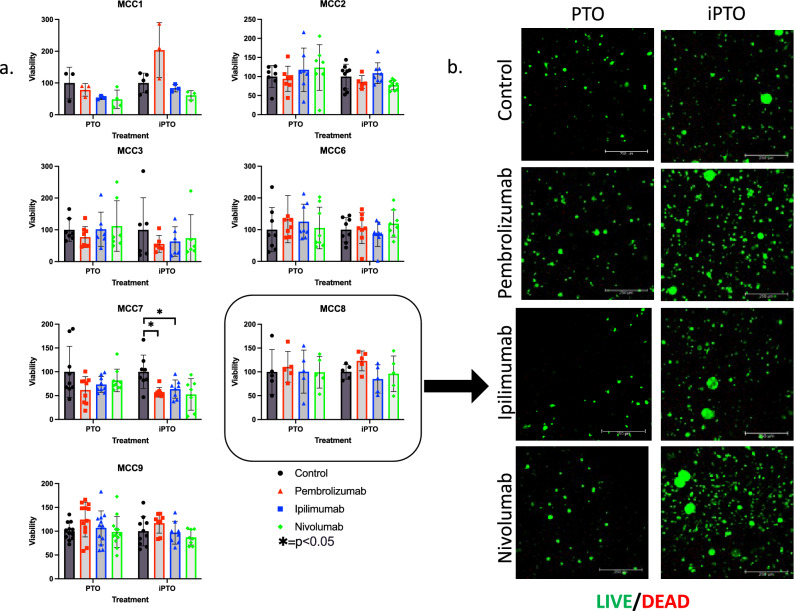
Demonstration of iPTOs in immunotherapy testing applications. (**a**) iPTO sets were treated with immunotherapies and viability was recorded with CellTiter-Glo 3D. No iPTOs were determined to have significant response of < 50% viability compared to control, statistical significance of p < 0.05 compared to iPTO control and to PTO counter treatment to any immunotherapies tested. (**b**) LIVE/DEAD Panel for MCC8. Scale bars represent 250 microns. *p < 0.05.

**Figure 6:**
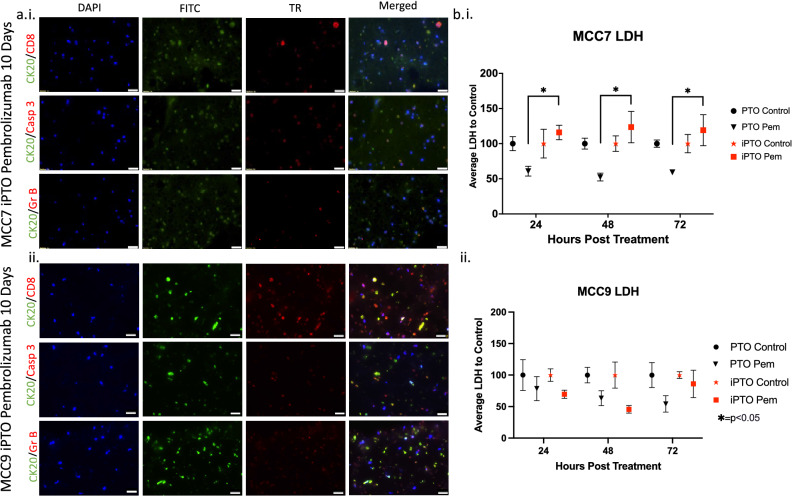
Comparative analysis of tumors temporally separated in the same patient. (**a**) Immunohistochemistry of iPTOs demonstrates robust CD8+ t-cell populations but low granzyme B mediated killing of CK20+ cells. (**b**) LDH cellular stress analysis determines an increase in pembrolizumab MCC7 iPTO set but not in MCC9, suggesting acquisition of resistance between the two tumors in this patient. All images taken at 40X magnification with scale bar 20 μM. *p < 0.05.

To effectively study the mechanisms behind immunotherapy efficacy, we incorporated immune cells isolated from whole blood or normal lymph node tissue into our PTO cultures to form immune enhanced patient tumor organoids (iPTOs). After seven days in culture, immunotherapy treatment was performed using three treatments approved for MCC: pembrolizumab, ipilimumab, and nivolumab. Based on our criteria for iPTO treatment efficacy of < 50% iPTO viability compared to control with statistical significance towards the matched iPTO control and the treatment matched PTO, ATP viability assays and confocal microscopy did not confirm significant immunotherapy effect on our immune organoids for any of the seven iPTO sets tested, with post treatment viability range of (55–110%) for pembrolizumab, ipilimumab (63–108%), and for nivolumab (74–120%) (Fig. 5). IHC analysis of treated organoids demonstrated minimal immune-mediated cytotoxicity measured by granzyme B and caspase 3 activity in tumor cells while demonstrating the presence of CD8+ T-cells over the course of the study period when compared to controls (Fig. 6a), Supplemental Figs. S3, S4, S5). Measuring a marker of cellular stress and tissue damage, LDH, levels at multiple timepoints post-treatment for MCC7 and MCC9 displayed lower levels of LDH at 24 and 48 h for MCC9, suggesting iPTO resistance to therapy early during treatment (Fig. 6bi, ii).

### MCC iPTOs demonstrate clinical correlation with their matched patients

Incorporating PTOs into a pre-clinical model requires clinical correlation. A total of four surgical specimens were obtained longitudinally from 2 patients, at different time points during their treatment course with pembrolizumab (Table 1). The first patient (MCC3) initially presented with a primary lesion of the face with additional concerning areas metastatic tumor activity in the mediastinum and pelvis on PET/CT imaging. This patient underwent resection of the primary MCC of the face and started adjuvant pembrolizumab, undergoing 4 infusions prior to passing from disease with corresponding iPTOs exhibiting a post treatment viability of 56% (Fig. 3).

The second patient (iPTO sets MCC2, MCC7, MCC9), developed a face recurrence (MCC2), 10 years post initial MCC extremity resection. PET/CT demonstrated new areas of metastasis at multiple sites in the face and neck. Seven cycles of adjuvant pembrolizumab resulted in a mixed clinical response to treatment. More specifically, response was observed at the neck metastatic lesions while the patient developed progression of disease with a new in transit face lesion that was resected for local control while the patient was maintained on treatment (MCC7). Two additional cycles of pembrolizumab infusions did not prevent development of a new rapidly growing face tumor (MCC9) that was also resected. Comparison of PTO chemotherapy treatments demonstrated increased resistance towards most single agent treatments and combinational regimens (Fig. 3). MCC2 iPTOs did not demonstrate patient sensitivity towards pembrolizumab (86% post treatment viability). The recurrent lesion (MCC7) demonstrated a viability of 57% post-pembrolizumab and an increase in LDH at 24-, 48- and 72-h post-treatment for pembrolizumab (Figs. 5a, 6bi). Finally, MCC9 did not demonstrate sensitivity towards pembrolizumab with a post-treatment viability of 119%, decreased levels of LDH, and lack of granzyme B and caspase 3 activity (Figs. 3, 5a, 6bii). Expression of granzyme B and caspase 3 in treated PTO CK20+ tumor cells decreased when comparing iPTO set MCC2 to later sets MCC7 and MCC9 (Supplementary Fig. S5).

## Discussion

Progress in MCC research has long been hindered by lack of tissue models and rarity of disease. Herein, we describe the creation of PTOs from resected surgical specimens without cell expansion that allows for operational availability and rapid testing within a week from generation. Multiple chemotherapy and immunotherapy regimens were compared for cytotoxic efficacy, demonstrating the potential in selecting both personalized treatments for the individual patient and the ability to scale for therapeutic screening in preclinical trials. Furthermore, the clinical response to immunotherapy of four MCC specimens derived from two patients was concordant with the response exhibited by their corresponding iPTOs. The optimization of these PTOs can increase our understanding of this rare cancer and possibly improve selection of systemic treatments.

Since the first development of PTOs, it has been theorized they may serve as a comparative model for patient therapy selection in addition to their ability as a research model for new therapies. Indeed, multiple studies have demonstrated high positive and negative predictive value for PTOs from a variety of tumor types^21–24^. Although assays correlating PTO and clinical response vary, a substantial decrease in PTO viability or growth has been routinely used as a threshold for cytotoxic efficacy and correlation. By utilizing a stringent threshold of 50% viability reduction to determine efficacy, we have shown both positive and negative PTO correlations in previous studies on melanoma and appendiceal cancer^15,18^. In this study, we also assessed viability via confocal microscopy and LDH content, but these were used only as supporting assays due to low throughput. iPTO correlation with clinical response was observed in four specimens previously treated with adjuvant pembrolizumab, a PD-1 inhibitor. iPTO sets developed from non-responding resected specimens demonstrated the presence of CD8+ cells with low levels of Granzyme B, post treatment cellular viability > 50% and decreased LDH levels suggesting low cytotoxic activity within the iPTOs despite the presence of check point inhibitors. The herein presented organoid results are artificially skewed towards to lack of immunotherapy response because corresponding patients were failing on immunotherapy and surgery was performed only to obtain local control. Therefore, these results cannot be used to suggest immunotherapy is not working for Merkel cell tumors. A multi-center phase 2 study observed a 56% response rate (14/25) amongst patients treated with pembrolizumab^25^. The mixed response to immunotherapy that was observed clinically along with the decrease in expression of these histologic markers in resected lesions progressing on immunotherapy is an indication of clonal evolution with co-existing malignant clones exhibiting differential responses to treatment. This is a clear demonstration that any response exhibited by the PTOs is reflective only of the biopsied lesions used to develop the organoids and not necessarily representative of the overall response of the patient in cases where tumor clonality exists in spatially distinct tumors.

Immunotherapy for MCC was first approved in 2017, and since then several agents have been developed and shown to improve patient survival^25–27^. Impaired immune function is related with increased MCC prevalence, therefore^28^ improving immune recognition of tumor cells may provide substantial benefits for treated patients^29^. The Merkel cell polyomavirus is identified in about 80% of patients, creating both a pro-oncogenic and anti-apoptotic signaling in affected cells^30,31^. By displaying a reliable matched expression of MCPyV in both excised tumors and matched PTOs, the presented organoid platform may prove to be useful to study and develop antiviral vectors or MCC vaccine development in cases where patient matched antigen presenting cells are incorporated in the co-cultured conditions^7,18^.

The herein reported PTO establishment rate of 89% aligns with other large studies using more common cancers such as colon and breast^21,32^ (Table 1). Our study utilizes a highly characterized hydrogel capable of controlled crosslinking providing consistent and reproducible results capable of supporting primary tissue cells without the need of xenogeneic factors. Further work with PTOs may allow for the creation and deployment of hydrogels with highly customized compositions to match the tissue of interest. The 10-day long workflow for organoid biofabrication described to generate chemosensitivity and immunosensitivity results, presents a translational research tool capable of seamless integration with a patient’s clinical needs and upon validation could potentially change the landscape of how clinicians navigate treatment decisions, from cohort analysis to personalized decisions. This is especially important for rare tumors where data from clinical trials are lacking. Lastly, PTOs enabled us to evaluate several chemotherapy combinations for synergism (Fig. 3). Patients might be spared unnecessary toxicity if combinational regimens can be singled out to the most effective drug in cases where synergism is not observed. The above work demonstrates the importance of diverting tissue from traditional “dead” tissue tumor banks into living tissue biorepositories.

While we were able to create and test a novel PTO model for MCC, there remain several limitations to our research. The modest power of the study is a direct effect of the rarity of MCC. Clinical correlation was not possible with all specimens as a number of patients had contraindications to receive systemic treatment including immunotherapy^5^. The presented clinical correlation describes negative predictive value, as many resected specimens originated from tumors clinically resistant to systemic treatment. However, it is logical that the survival of patients is ultimately determined by non-responding clones therefore identification of non-responders is important in avoiding ineffective treatments as well as associated treatment related toxicity and cost. Establishing positive predictive value will be a necessary endpoint in future studies. Finally, although radiation is a potential treatment options for MCC, we did not incorporate this treatment parameter in our study as previously described in other primaries^33^.

Despite the promises of PTOs in MCC and other rare cancers research, organoids are not yet ready to be applied in clinical practice without sufficiently powered correlative data with patient outcomes. Introducing of organoids as correlative tools in prospective randomized clinical trials is the way to move forward along with extensive genomic and proteomic characterization. Routine establishment and immune enhancement of MCC PTOs is feasible directly from resected surgical specimens allowing for personalized research and exploration of treatment regimens in the preclinical setting.

## Methods

All methods were performed under institutional guidelines in accordance with Wake Forest Baptist Health policies.

### Tumor procurement and processing

Tissue, blood, and lymph node specimens were obtained from informed consenting adult patients with MCC undergoing resection between December 2018 and January 2022. Specimens were obtained under an IRB protocol approved by the Wake Forest Human Research Protection Program. Specimens were placed in Roswell Park Memorial Institute (RPMI) media and transferred to the Wake Forest Organoid Research Center (WFORCE) for processing within a post-operative 2-h framework.

Upon tissue receival, tumors were washed in phosphate-buffered saline with 100 U/mL penicillin–streptomycin, 5 mg/mL gentamicin, and 5 mg/mL amphotericin B for two 5-min cycles. When possible, a portion of each specimen was removed and utilized for histology; this was performed if tissues were sufficiently large to prepare organoid studies. Specimens were minced and placed in a 15 mL conical in a 3 mL solution of Dulbecco’s Modified Eagle’s Medium (DMEM) with 100,000 CDA units/mL collagenase HA (001-1050; VitaCyte, Indianapolis, IN), 22,000 units/mL protease (003-1000; VitaCyte), and 200 U/mL DNAse 1 (07470; STEMCELL, Cambridge, MA) per gram of tissue for up 60 min under agitation at 37 °C. Upon complete tissue dissolution, enzymatic digestion was terminated with cold culture medium containing FBS and resultant tumor solution was filtered through a vacuum filtration kit (SCNY0060; Millipore Sigma, Burlington, MA), then centrifuged to isolate the cell pellet. Supernatant was removed and the cell pellet resuspended with Red Blood Cell Lysis Buffer (Abcam, Cambridge, UK) according to company protocol. Lysis buffer was removed, and cells were counted using a NucleoCounter NC-200 (Chemometec, Denmark). Whole blood was obtained from patients for retrieval of lymphocytes using Ficoll-Paque PLUS and corresponding protocol (GE Healthcare, Chicago, US). A normal lymph node from one patient was obtained for iPTO fabrication. Lymph nodes were processed similarly to whole tissue as described above.

### Organoid fabrication and culture

The tumor cell pellet was resuspended with a thiol-modified hyaluronan/heparin (Heprasil; Advanced Biomatrix, San Diego, CA) prepared to manufacturer’s instructions with 0.1% w/v Irgacure 2959 (5200, Advanced BioMatrix, San Diego, CA) added as a photo-initiator and 3 mg/mL methacrylated collagen (PhotoCol; Advanced Biomatrix) solution in a 1:3 volume ratio at a cell density of 10 million cells/mL. Patient-derived tumor organoids (PTOs) were created by seeding 5 µL of the hydrogel/cell mixture into individual wells of a 96-well non-tissue culture treated plate and photocrosslinked by exposure to ultraviolet light (365 nm, 18 W/cm^2^) from a BlueWave 75 V.2 UV spot lamp (Dymax Corp., Torrington, CT) for 1 s to crosslink organoids. PTOs were cultured in 200 mL media containing DMEM-F12 with 5% FBS, 1% penicillin–streptomycin, 1% l-glutamine, 50 ng/mL EGF (PHG 0313; ThermoFisher Scientific), 10 µM Y-27632 (S1049, Selleckchem, Houston, TX) with media changed every 3–4 days.

Immune-enhanced PTOs (iPTOs) were created by combining the immunocompetent cells from matched patient whole blood (blood iPTOs) or nodal lymph tissue (lymph iPTOs) in a 3:1–5:1 ratio according to cell yield with tumor cells and seeded onto plates as described above. The organoids in addition to tumor and CD8^+^ cells, contain CD4^+^ and APC cells, as well as stroma as described previously^15,18^. Organoids were cultured for 7 days prior to treatment.

### Therapeutic screening

Organoids were subsequently treated after 7 days of culture. Chemotherapies included cisplatin (1, 10, 100 µM) (232120, Sigma Aldrich), doxorubicin (0.1, 1, 10 µM) (S1208, Selleckchem), vincristine (0.1, 1, 10 µM) (V8879 Sigma Aldrich), doxorubicin with cisplatin (0.1/1, 1/10, 10/100 µM), cisplatin with etoposide (S1125, Selleckchem) (1/0.1, 10/1, 100/10 µM) (Selleckchem), and doxorubicin with vincristine (0.1/0.1, 1/1, 10/10 µM). For immunotherapy treatment, 100 nM of pembrolizumab (A2002, Selleckchem), ipilimumab (A2001, Selleckchem) or nivolumab (A2005, Selleckchem) was used on both iPTOs and PTOs in parallel treatments. Media was removed from the wells and drug solutions mixed in culture media were added. Organoids remained in treated media solution for 72 h prior to endpoint viability assessment.

### Organoid viability assessment

After 3 days of incubation in drug-containing media, organoids were assessed with LIVE/DEAD staining, CellTiter-Glo 3D, and LDH-GLO cytotoxicity viability assays. LIVE/DEAD staining (L3224; Invitrogen, Carlsbad, CA) was performed according to the manufacture’s protocol and incubated at 37 °C for 2 h prior to imaging. Fluorescent imaging was performed using a Leica TCS LSI macro confocal microscope (Leica Microsystems Inc., Buffalo Grove, IL). Images from red and green channels were overlaid and stacked in maximum projection.

Quantitative viability was assessed utilizing CellTiter-Glo 3D Cell Viability (G968B; Promega, Madison, WI) and LDH-GLO cytotoxicity assays (J2381; Promega). LDH (Lactose Dehydrogenase) is an enzyme which catalyzes multiple necessary steps in the formation of energy for the cell. Release of LDH is for marker of cellular stress and can be clinically utilized to measure tissue damage. By observing small increases in this marker, it is feasible to ascertain these cells are not undergoing stress or cell damage, which can be utilized as a non-lytic measurement of cell damage as opposed to CellTiter-Glo 3D, at the cost of decreasing assay throughput. For CellTiter-Glo 3D Cell Viability assay, half the media (100 mL) was removed from individual wells and 100 mL of assay reagent was added to each well, followed by incubation at room temperature on a shaker for 30 min. LDH-GLO cytotoxicity assay was performed by preserving culture medium from post-treatment days 1, 2, 3 and running the assay on the same day according to manufacturer specifications. Well contents for both assays were transferred to a Costar White Polystyrene 96 well Assay Plate (3912; Corning, NY) and analyzed with a Veritas Microplate Luminometer (Turner BioSystems, Sunnyvale, CA).

### Organoid tissue characterization

Organoids were fixed for histology on day 10 of culture in 4% paraformaldehyde for 4 h. Organoids were processed, paraffin embedded, and sectioned at 5-μm intervals for staining. Organoid and tissue sections were stained with hematoxylin and eosin (H&E).

Chromogenic immunohistochemistry was performed to identify PTO and tissue cell populations. Antigen retrieval was performed on unstained slides using pH 6 citrate buffer. Tissue and organoids were incubated with Cytokeratin 20 (CK20, ab76126, Abcam), Pan-Cytokeratin (Pan-CK, NBP2-29429, NOVUS, Centennial, CO), Neurofilament (NFH, 13-1300, Thermofisher, Waltham, MA), Synaptophysin (SYP, MA5-14532, Thermofisher), Chromogranin A (ChgA, ab15160, Abcam), and Merkel Cell Polyomavirus T-Antigen (MCPyV, MABF2316, Millipore Sigma) primary antibodies overnight. Appropriate biotinylated secondary antibody (abcam) incubation followed for 1 h. Slides were then exposed to DAB solution (SK-4105, Vector Laboratories, Burlingame CA, USA) for two minutes and counterstained with hematoxylin. Imaging was performed on an Olympus BX-63 microscope (Olympus, Tokyo, Japan).

Additional staining was performed with fluorescent immunohistochemistry (IHC) to characterize iPTO immunotherapy treatment response. Unstained slides underwent antigen retrieval in a pH 6 citrate buffer solution prior to blocking with Dako Protein Block for 30 min. Fluorescent IHC was performed with antibodies for cleaved caspase 3 (9661S, Cell Signaling Technologies, Danvers MA), CD-8 (ab4055, abcam), CK-20 (MA5-13263, Invitrogen), and granzyme B (ab4059, abcam) to slides in ratios of 1:500, 1:200, 1:200, 1:100, 1:400, respectively. After incubation overnight at 4C, appropriate species reactive secondary Alexa Fluor 488 or Alexa Fluor 594 antibodies (Biotium, Fremont, CA) were applied to samples for 1 h at a 1:1000 dilution. Sections were then incubated with DAPI for 5 min prior to finalization with coverslipping. An Olympus BX-63 upright fluorescent microscope was used to image the sections. Pixel quantification for fluorescent imaging was performed using ImageJ FIJI software.

### Definition of organoid treatment response

There is no consensus regarding what represents therapeutic efficacy in organoids. Herein, we utilize a conservative approach for considering an organoid to be responsive to therapy, consisting of three distinct criteria that simultaneously must be met by iPTOs: (1) demonstrate a statistically significant reduction in cell viability when compared to untreated (control) organoids (Ex: iPTO control vs iPTO treated), and (2) demonstrate a statistically significant reduction in cell viability when comparing treated organoids from immune enhanced to the non-immune enhanced counter conditions (Ex: immunotherapy treated iPTO vs immunotherapy treated PTO), and (3) exhibit a post immunotherapy CellTiter-Glo 3D viability < 50%.

Similarly, non-immune enhanced PTOs must achieve two criteria: (1) demonstrate statistically significant reduction in viability compared to untreated PTO control organoids and (2) exhibit a post therapy CellTiter-Glo 3D viability reduction of > 50%. We arbitrarily selected 50% viability reduction as the lowest threshold suggestive of therapy response. This number can be changed based on the desired tumor response in need to be studied or the kinetics and the tumor biology of every individual patient, demonstrating the plasticity of the platform.

### Statistical analysis

All data is expressed as mean ± standard deviation for each experimental group. Each condition combination consisted of 3 or more organoids for analysis. CellTiter-Glo 3D assay values of treated organoids were standardized to condition-matched (iPTO or PTO) controls prior to statistical analysis. Two sample t-tests were used to assess whether cell viability was different between desired comparison groups. A rigorous threshold was chosen for determining whether a treatment group showed response to therapy. This threshold requires all treatment response conditions (identified in the previous section) to be met to consider an organoid as being a treatment response. This approach was used to diminish the probability of a type 1 error occurring. Specifically, the chance that two t-tests described above would both be significant at p < 0.05 (rather than because both indicated evidence of a treatment response) would be 0.25%. Further, if the probability that post immunotherapy CellTiter-Glo 3D viability of less than 50% were a random event (i.e., 50% chance that it would occur by chance) then the combined probability that all three events would occur simultaneously by chance would be 0.125% or 12.5 in 10,000 for immunotherapy studies. Drug screen studies were determined to be successful for a patient if untreated control PTOs demonstrated adequate viability at day 10 of culture, which coincided with termination of drug screens. Adequate viability is described as blank value < 1% of control condition. Statistical analysis was performed with GraphPad Prism (GraphPad Software Inc., USA).

## Supplementary Information

Supplementary Figures.

## Data Availability

Datasets generated and/or analyzed during the current study are not publicly available due to risk of patient identifying information based on the rareness of disease but are available from the corresponding author on reasonable request.
