# Supplementary material for: Application of immune enhanced organoids in modeling personalized Merkel cell carcinoma research
Source: Sci Rep. 2022 Aug 16;12:13865. doi: 10.1038/s41598-022-17921-6 (PMC9380677; doi:10.1038/s41598-022-17921-6)
Supplement: Supplementary file 1 — Supplementary Figures. [file 41598_2022_17921_MOESM1_ESM.pdf]

Supplemental Figures

Application of Immune Enhanced Organoids in Modeling Personalized Merkel Cell Carcinoma Research

Steven D. Forsythe[1,2,3], Richard A. Erali,[3,4,5], Preston Laney[1,3], Hemamylammal Sivakumar[1,6], Wencheng Li MD[7], Aleksander Skardal[1,2,6,8], Shay Soker[1,2,3,5], Konstantinos I. Votanopoulos[1,2,3,4,5] *

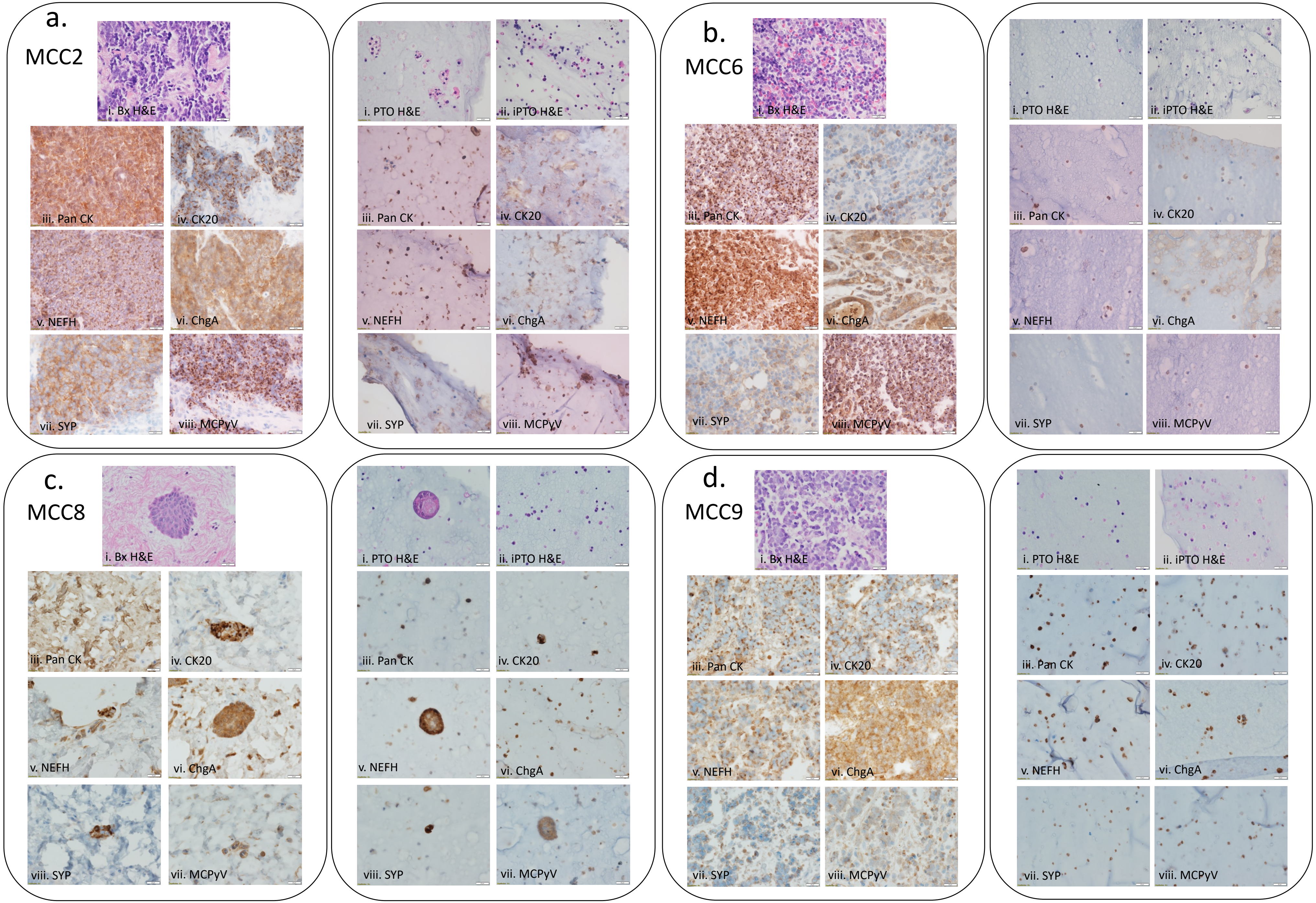
a.
MCC2
i. Bx H&E
iii. Pan CK
iv. CK20
v. NEFH
vi. ChgA
vii. SYP
viii. MCPyV
i. PTO H&E
ii. iPTO H&E
iii. Pan CK
iv. CK20
v. NEFH
vi. ChgA
vii. SYP
viii. MCPyV
b.
MCC6
i. Bx H&E
iii. Pan CK
iv. CK20
v. NEFH
vi. ChgA
vii. SYP
viii. MCPyV
i. PTO H&E
ii. iPTO H&E
iii. Pan CK
iv. CK20
v. NEFH
vi. ChgA
vii. SYP
viii. MCPyV
c.
MCC8
i. Bx H&E
iii. Pan CK
iv. CK20
v. NEFH
vi. ChgA
viii. SYP
vii. MCPyV
i. PTO H&E
ii. iPTO H&E
iii. Pan CK
iv. CK20
v. NEFH
vi. ChgA
viii. SYP
vii. MCPyV
d.
MCC9
i. Bx H&E
iii. Pan CK
iv. CK20
v. NEFH
vi. ChgA
vii. SYP
viii. MCPyV
i. PTO H&E
ii. iPTO H&E
iii. Pan CK
iv. CK20
v. NEFH
vi. ChgA
vii. SYP
viii. MCPyV

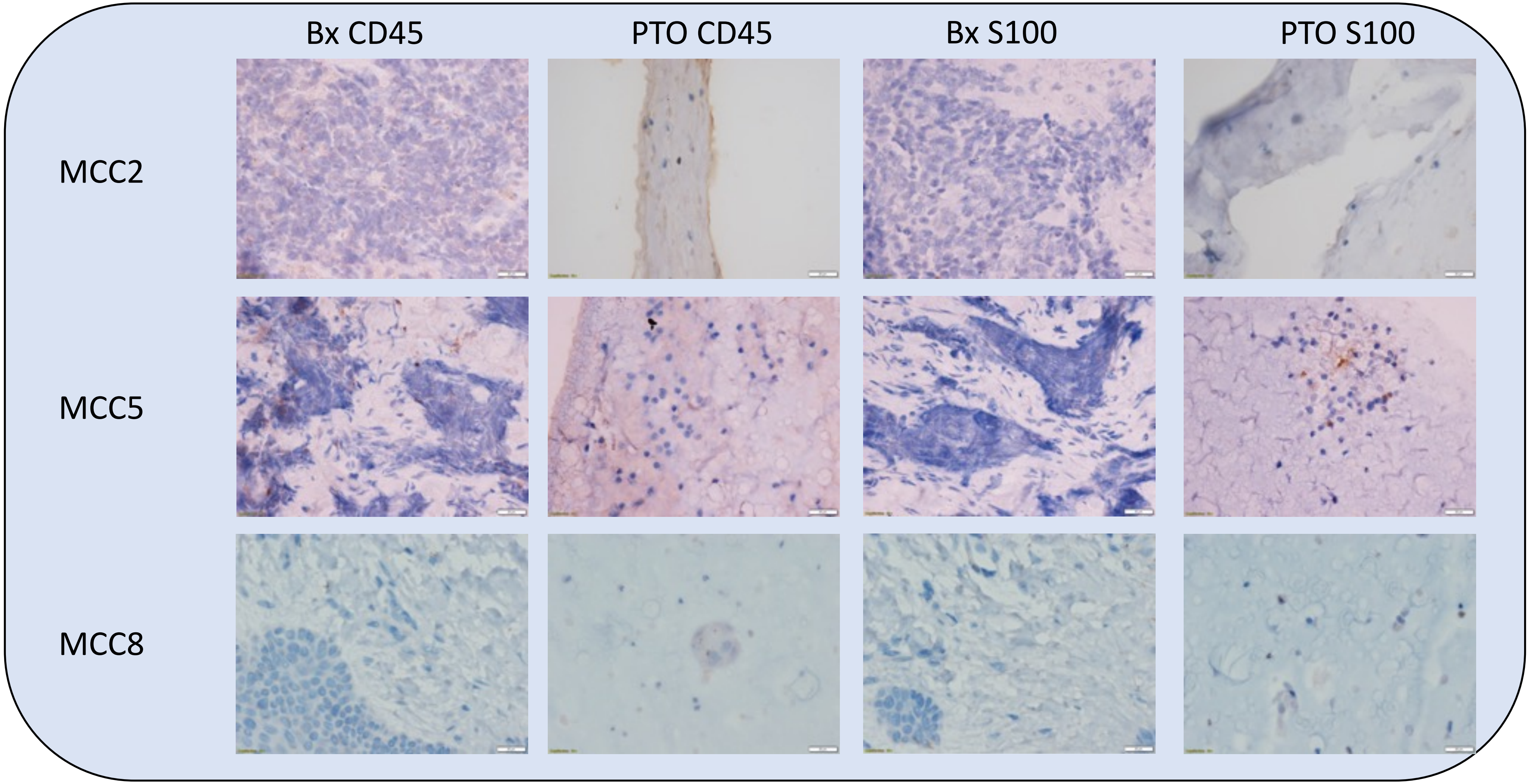
Bx CD45
PTO CD45
Bx S100
PTO S100
MCC2
MCC5
MCC8

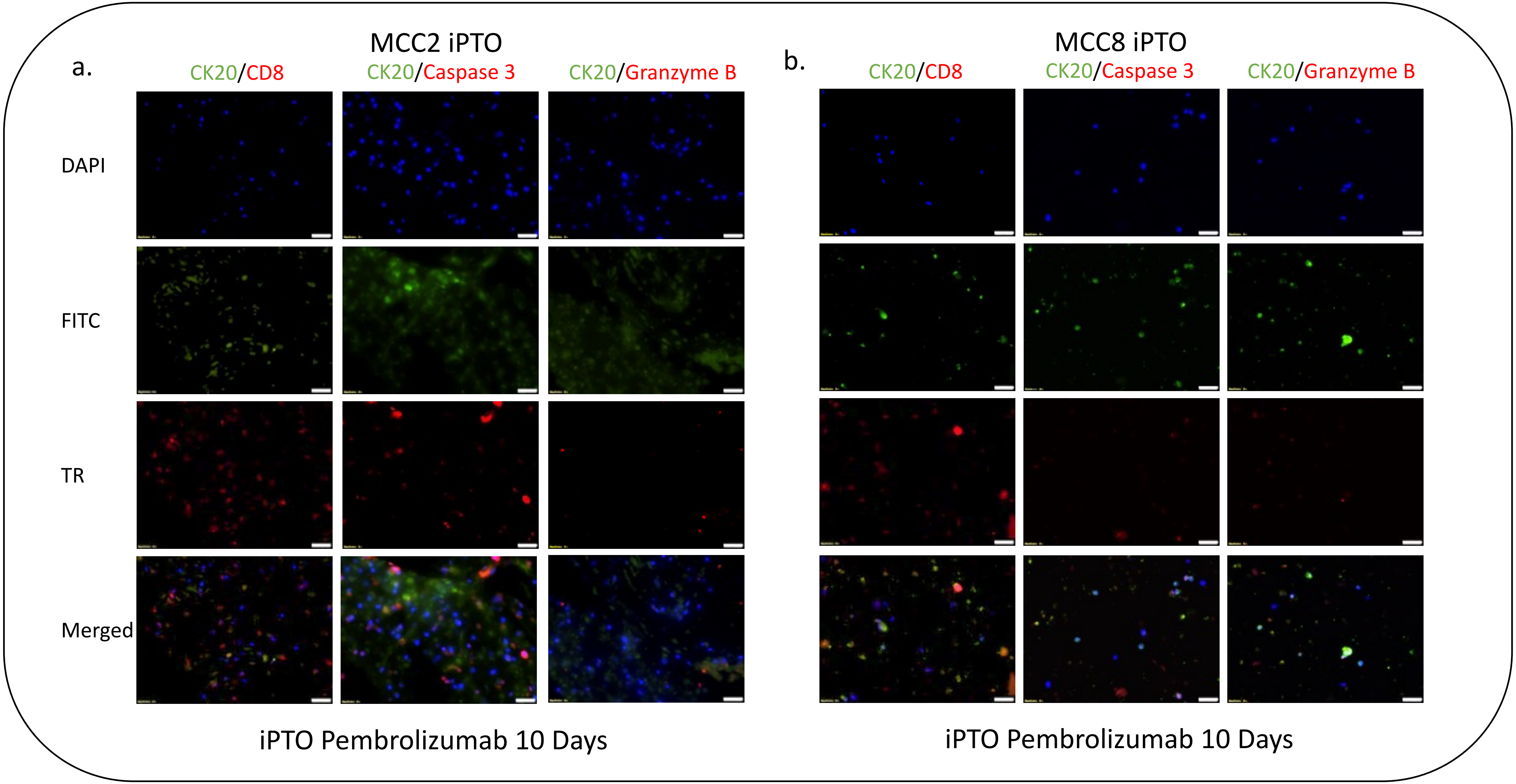
a.
MCC2 iPTO
CK20/CD8
CK20/Caspase 3
CK20/Granzyme B
DAPI
FITC
TR
Merged
iPTO Pembrolizumab 10 Days
b.
MCC8 iPTO
CK20/CD8
CK20/Caspase 3
CK20/Granzyme B
iPTO Pembrolizumab 10 Days

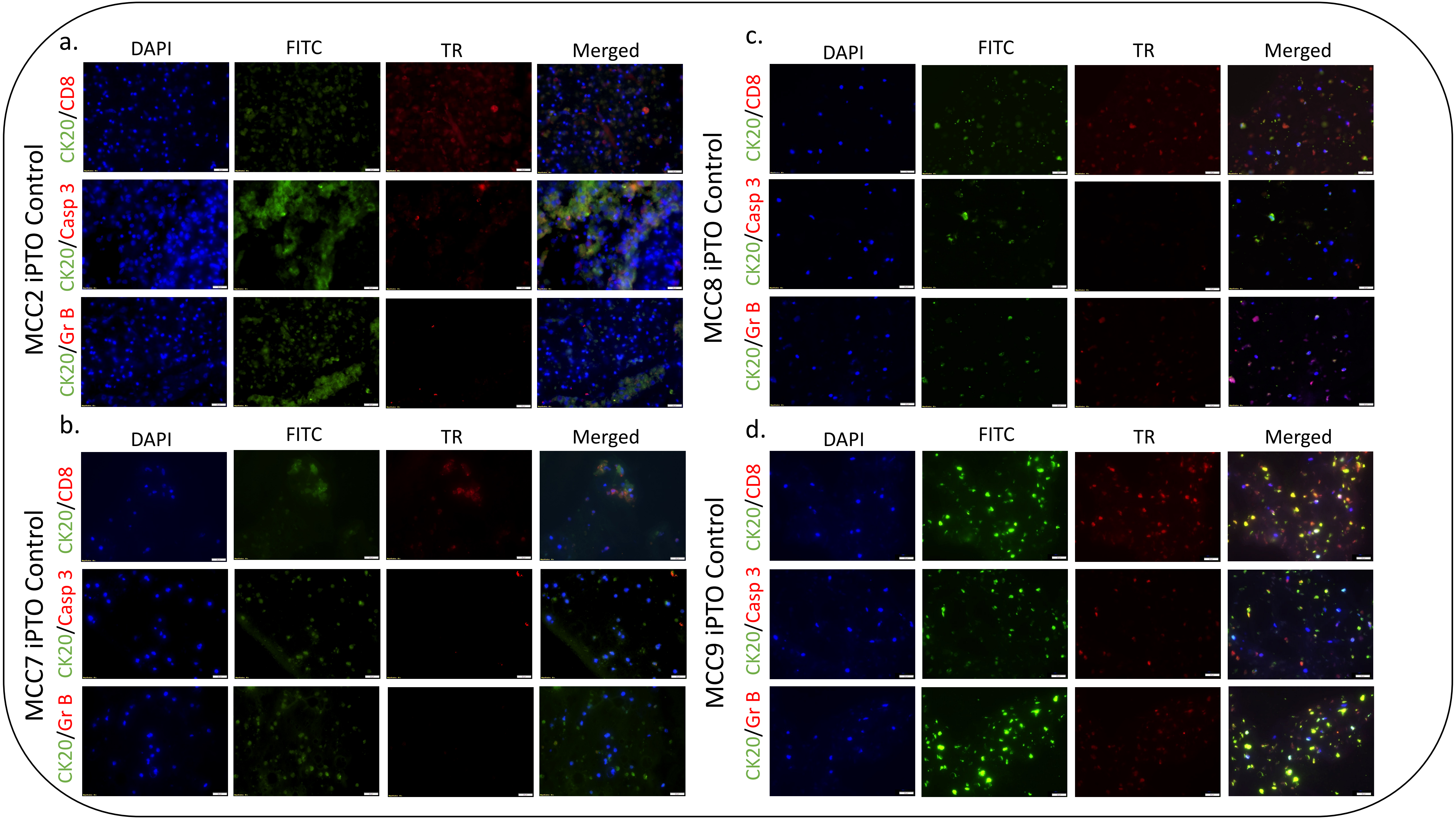
a.
DAPI
FITC
TR
Merged
MCC2 iPTO Control
CK20/CD8
CK20/Casp 3
CK20/Gr B
b.
DAPI
FITC
TR
Merged
MCC7 iPTO Control
CK20/CD8
CK20/Casp 3
CK20/Gr B
c.
DAPI
FITC
TR
Merged
MCC8 iPTO Control
CK20/CD8
CK20/Casp 3
CK20/Gr B
d.
DAPI
FITC
TR
Merged
MCC9 iPTO Control
CK20/CD8
CK20/Casp 3
CK20/Gr B

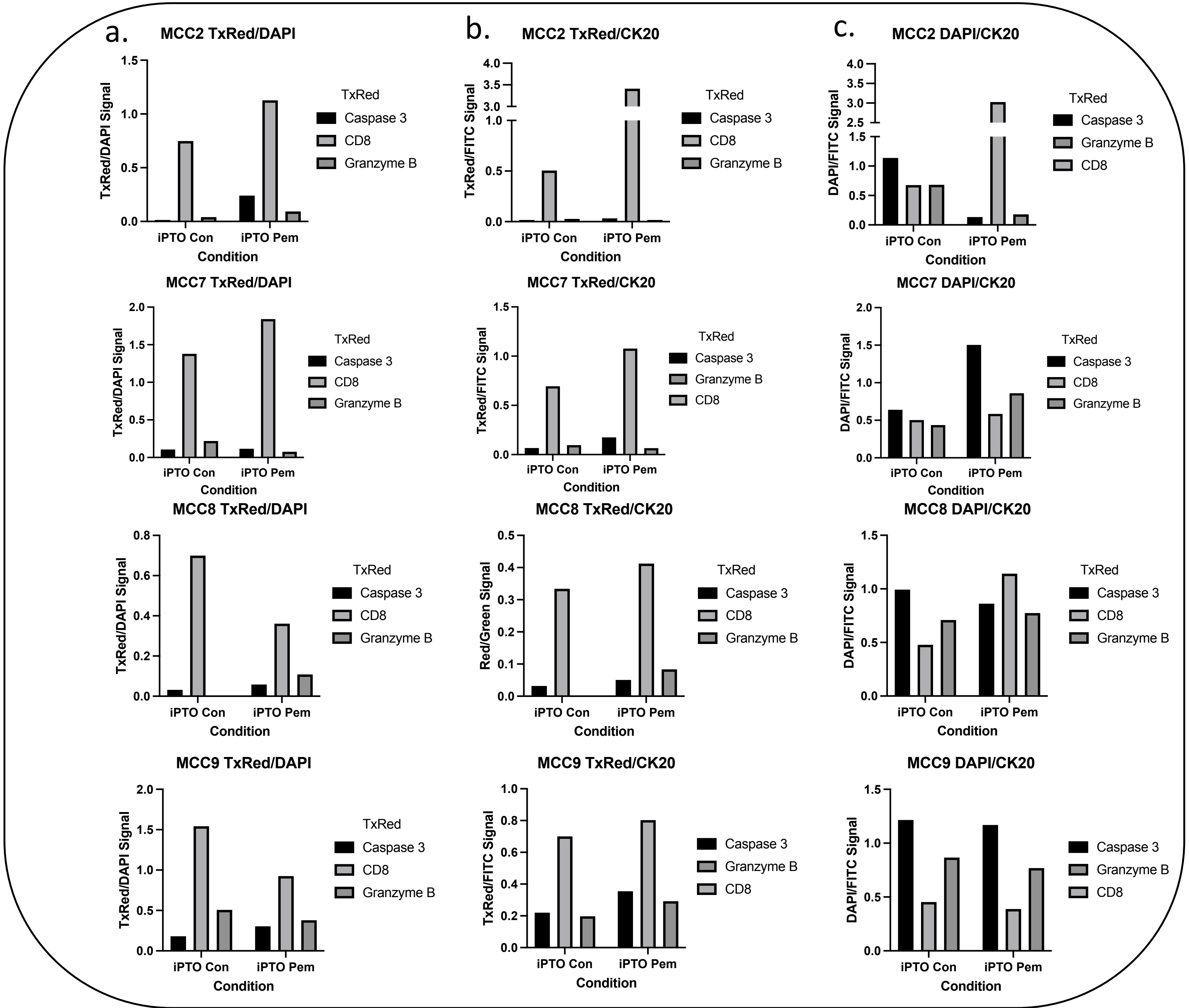
a.
b.
c.
MCC2 TxRed/DAPI
TxRed/DAPI Signal
1.5
1.0
0.5
0.0
iPTO Con
iPTO Pem
Condition
TxRed
Caspase 3
CD8
Granzyme B
MCC2 TxRed/CK20
TxRed/FITC Signal
4.0
3.5
3.0
1.0
0.5
0.0
iPTO Con
iPTO Pem
Condition
TxRed
Caspase 3
CD8
Granzyme B
MCC2 DAPI/CK20
DAPI/FITC Signal
4.0
3.5
3.0
2.5
1.5
1.0
0.5
0.0
iPTO Con
iPTO Pem
Condition
TxRed
Caspase 3
Granzyme B
CD8
MCC7 TxRed/DAPI
TxRed/DAPI Signal
2.0
1.5
1.0
0.5
0.0
iPTO Con
iPTO Pem
Condition
TxRed
Caspase 3
CD8
Granzyme B
MCC7 TxRed/CK20
TxRed/FITC Signal
1.5
1.0
0.5
0.0
iPTO Con
iPTO Pem
Condition
TxRed
Caspase 3
Granzyme B
CD8
MCC7 DAPI/CK20
DAPI/FITC Signal
2.0
1.5
1.0
0.5
0.0
iPTO Con
iPTO Pem
Condition
TxRed
Caspase 3
CD8
Granzyme B
MCC8 TxRed/DAPI
TxRed/DAPI Signal
0.8
0.6
0.4
0.2
0.0
iPTO Con
iPTO Pem
Condition
TxRed
Caspase 3
CD8
Granzyme B
MCC8 TxRed/CK20
Red/Green Signal
0.5
0.4
0.3
0.2
0.1
0.0
iPTO Con
iPTO Pem
Condition
TxRed
Caspase 3
CD8
Granzyme B
MCC8 DAPI/CK20
DAPI/FITC Signal
1.5
1.0
0.5
0.0
iPTO Con
iPTO Pem
Condition
TxRed
Caspase 3
CD8
Granzyme B
MCC9 TxRed/DAPI
TxRed/DAPI Signal
2.0
1.5
1.0
0.5
0.0
iPTO Con
iPTO Pem
Condition
TxRed
Caspase 3
CD8
Granzyme B
MCC9 TxRed/CK20
TxRed/FITC Signal
1.0
0.8
0.6
0.4
0.2
0.0
iPTO Con
iPTO Pem
Condition
Caspase 3
Granzyme B
CD8
MCC9 DAPI/CK20
DAPI/FITC Signal
1.5
1.0
0.5
0.0
iPTO Con
iPTO Pem
Condition
Caspase 3
Granzyme B
CD8

Supplementary Figure Legends

Supplementary Figure 1- Additional IHC staining on a) MCC2 patient tissue (Bx) and PTO set, b) MCC6 patient tissue and PTO set, c) MCC8 patient tissue and PTO set, and d) MCC9 patient tissue and PTO set, including i) cellular morphology (H&E), ii) iPTO cellular morphology (H&E), iii) pan cytokeratin (Pan CK), iv) cytokeratin 20 (CK20), v) neurofilament (NFH), vi) chromogranin A (ChgA), vii) synaptophysin (SYP and viii) Merkel cell polyomavirus (MCPyV). All images taken at 40X magnification with scale bar=20 μM

Supplemental Figure 2- Comparison of immunohistochemistry markers for alternate tumor diagnoses demonstrates lack of expression in MCC2, MCC5 and MCC8 for multiple myeloma positive marker CD45 and melanoma positive marker S100 in both tissue (Bx) and patient tumor organoids (PTOs). All images taken at 40X magnification with scale bar=20 μM

Supplemental Figure 3- Immunofluorescent staining for pembrolizumab treated iPTO sets a) MCC2 and b) MCC8. All images taken at 40X magnification with scale bar=20 μM

Supplemental Figure 4- Immunofluorescent staining for 10-day control iPTO sets a) MCC2 b) MCC7 c) MCC8 and d) MCC9. All images taken at 40X magnification with scale bar=20 μM

Supplementary Figure 5- Quantification for immunohistochemistry staining in control and treated iPTOs in comparing a) TxRed/DAPI signal, b) TxRed/FITC and c) DAPI/FITC. a) TxRed/DAPI demonstrates the quantification of TxRed markers (Caspase3, CD8, and Granzyme B) in reference to DAPI nuclear staining. b) TxRed/FITC demonstrates the quantification of TxRed markers (Caspase3, CD8, and Granzyme B) with CK20+ tumor cells tagged with FITC. c) DAPI/CK20 demonstrates the ratio of DAPI nuclear staining in reference to CK20+ tumor cells tagged with FITC.
